# Abstracts of the 6th Meeting of the Neapolitan Brain Group

**DOI:** 10.1186/s12868-018-0466-4

**Published:** 2018-11-13

## I1 The Neapolitan Brain Group

### M. Cataldi^1^, E. Di Schiavi^2^, C. Lucini^3^, E. Del Giudice^4^

#### ^1^Department of Neuroscience, University of Naples “Federico II”, Napoli, Italy; ^2^Institute of Bioscience and BioResources, IBBR, CNR, Napoli, Italy; ^3^Department of Veterinary Medicine and Animal Productions, University of Naples “Federico II”, Napoli, Italy; ^4^Department of Translational Medicine, Section of Pediatrics, University of Naples “Federico II”, Napoli, Italy

##### **Correspondence:** E. Del Giudice (endelgiu@unina.it)

*BMC Neuroscience* 2018,** 19(suppl 3)**:I1


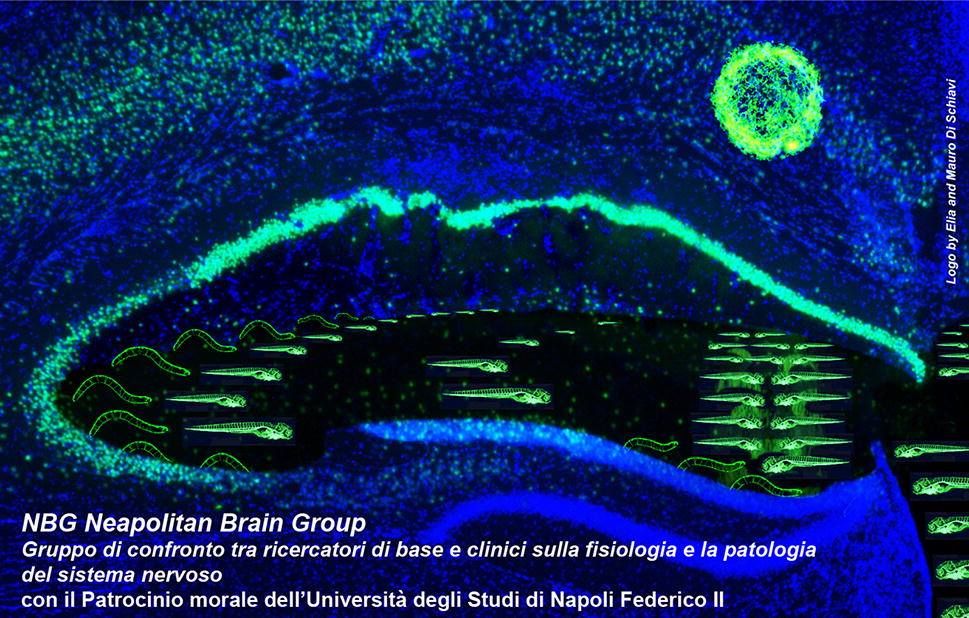
The Neapolitan Brain Group (NBG) is a discussion group including basic and clinical researchers of the Campania area (and, more generally, Southern Italy), interested in the study of the physiology and the pathophysiology of the diseases of the nervous system. The group was founded in 2015 by Professor Ennio Del Giudice of the University of Naples Federico II, Department of Translational Medical Sciences (Section of Pediatrics).

The group wants to provide an opportunity to meet, in an informal atmosphere, scientists from clinical and basic research who wish to improve their mutual knowledge and, as far as possible, explore fruitful collaborations.

NBG is open to all those who are interested in clinical and basic neurosciences, in particular young training people, such as doctoral students, postdocs, post-graduates, students and trainees of universities and other research institutions.

On June 4, 2015, February 4 and April 28, 2016 and December 15, 2016, the first five meetings were held with short communications, while on June 9, 2016 a monothematic meeting for general interest was organized on “Molecular, pathophysiological and clinical mechanisms in the neuroprotection of neonatal hypoxia “. In these meetings more than 90 scientists presented their research projects: among them basic and clinical researchers, PhD students, postdocs and specialists from all the Universities of Campania as well as from research Institutions such as CNR, TIGEM, SZN and CEINGE.

The NBG past meeting organizers were: Ennio Del Giudice (endelgiu@unina.it), Carla Lucini (carla.lucini@unina.it), Mauro Cataldi (mauro.cataldi@unina.it), Elia Di Schiavi (elia.dischiavi@ibbr.cnr.it).

**Acknowledgements**

The organizers wish to thank the SZN President Roberto Danovaro for kindly accepting to host the meeting and for supporting it, the Italian Society for Neuroscience (SINS) and “University of Naples Federico II” for patronage, Dr. Graziano Fiorito (SZN, Napoli) for support, the sponsors for coffee and lunch breaks and the precious collaboration of the Scientific Committee, in particular the external reviewers for selecting the abstracts: A. Cellerino (Scuola Normale Superiore di Pisa), E.M. Valente (Università di Pavia), M.A. Hilliard (QBI, Australia).

The Abstracts are presented on behalf of the Neapolitan Brain Group (NBG).

**Further information about the conference**

For more information please visit:

[http://www.neapolitanbraingroup.it/]

[https://www.facebook.com/NBG2000/]

## I2 The Stazione Zoologica *Anton Dohrn*

### P. Sordino^1^, B. Fantini^2^

#### ^1^Stazione Zoologica Anton Dohrn, Napoli, Italy; ^2^Faculty of Medicine, University of Geneva, Geneva, Switzerland

##### **Correspondence:** P. Sordino (sordino@szn.it)

*BMC Neuroscience* 2018, ** 19(suppl 3)**:I2


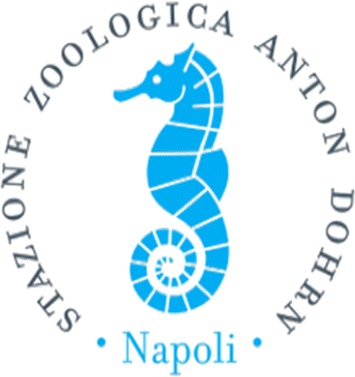
Anton Dohrn, founder of the Zoological Station, was born in Stettin, in Pomerania, now part of Poland, in 1840. He studied zoology and medicine but without much enthusiasm. His ideals changed when Ernst Haeckel introduced him to the works and theories of Charles Darwin. Dohrn became a fervent defender of Darwin’s theory of ‘descent with modification’, the theory of evolution by natural selection. He decided to devote his life to collecting facts and ideas in support of Darwinism, the starting point of a lifelong adventure. During his academic career, Dohrn worked at facilities located on the seashore. Here a project took shape to cover the globe with a network of stations of biological research where scientists could stop, collect material, realize observations and experiments, before moving to the next station. The choice of Naples was due to the biological richness of the Mediterranean Sea and the opportunity to develop research of great international importance in a large, internationally oriented city. He had thought that a public aquarium in one of Europe’s largest and most attractive cities, with a large influx of tourists, could earn enough to pay for a permanent assistant for the laboratories. Putting together imagination, willpower, diplomatic skills and a good dose of luck, thanks to the friendly support of scientists, artists and musicians, Anton Dohrn overcame doubt, ignorance and misunderstanding and was able to persuade the local authorities to give him a piece of land in the beautiful Royal Park, now Villa Comunale. In 1873 the first building of the Zoological Station was finished and the official opening took place in 1875. One important feature for the success of the institution was the remarkable agility and flexibility of its structure. It was an international institution by nature, founded by a German, managed as a family business and organized according to the German academic model, but localized in Italy, with an opening at the scientific and financial contributions of each country and institution. The idea of an agile, flexible, and small initiative but full of courage characterized the ‘spirit’ of the Stazione Zoologica Anton Dohrn, from its origins. In order to promote the international nature of the station and to ensure the freedom of research and its political and economic independence, Dohrn introduced a series of innovative measures, like renting work and research spaces for an annual fee, and selling samples and biological preparations. Also, Anton Dohrn donated his important library to the Zoological Station and requested donations to scientific publishers, academics and scientists, as Darwin and Huxley. The bibliographic collections of the Stazione Zoologica quickly became an invaluable instrument, still unmatched in Europe.

**ORAL COMMUNICATIONS**

## O1 Brain metabolic DNA is synthesized by reverse transcription in cytoplasmic organelles

### Bruno Rutigliano, Marina Prisco, Joyce Casalino, Carolina Cefaliello, Antonio Giuditta

#### Biology Department, Federico II University, Napoli, Italy

##### **Correspondence:** Antonio Giuditta (giuditta@unina.it)

*BMC Neuroscience* 2018, ** 19(suppl 3)**:O1

Brain metabolic DNA (BMD) is not involved in cell division or DNA repair but is modulated by learning, sleep memory processing, and circadian oscillations. Using routine methods of subcellular fractionation, newly synthesized BMD from male rats was shown to be localized in crude nuclear, mitochondrial, and microsomal fractions and in two fractions of purified nuclei. Sub-fractionation of the mitochondrial fraction also indicated its prevalent presence in free mitochondria, synaptosomes and myelin. Cesium density profiles of homogenate and subcellular BMD analyzed after short and longer incorporation times strongly suggested that BMD is synthesized by reverse transcription in cytoplasmic organelles before acquiring the double stranded DNA conformation and being transferred to nuclei. Kinetic analyses covering incorporation periods of several weeks provided evidence that subcellular BMD undergoes a massive turnover that depends on rat age. Recent immunofluorescence data of mouse BrdU-labeled BMD have confirmed its cytoplasmic localization and reverse transcription.

Data suggest that BMD might act as a temporary store of cellular responses to environmental changes that could be used in forthcoming experiences. The view supports the concept of a somatic genome that is dynamically modulated by adaptation to the environment.

## O2 Nerve regeneration in the cephalopod mollusc *Octopus vulgaris*: from images to discoveries

### Pamela Imperadore^1,2^, Katherina Orellana^3^, Graziano Fiorito^1^

#### ^1^Department of Biology and Evolution of Marine Organisms, Stazione Zoologica Anton Dohrn, Napoli, Italy; ^2^Association for Cephalopod Research - CephRes, Napoli, Italy; ^3^Life Science Research, Leica Microsystems, Milano, Italy

##### **Correspondence:** Pamela Imperadore (imperadore.p@gmail.com)

*BMC Neuroscience* 2018, ** 19(suppl 3)**:O2

Pallial nerve lesion determines interruption of the circuitry connecting the central nervous system and the periphery, resulting in paralysis of *Octopus vulgaris* mantle muscles supporting breathing and in the loss of the central control of body patterning, at the level of the denervated area. Complete functional recovery occurs at about 30 days for normal ventilation, and about 2 months for reinstating the full control of body pattern.

To disclose cellular and structural events occurring in the injured nerves we utilized a combination of approaches including multiphoton microscopy, that we applied to cephalopods tissues for the first time. These allow label-free ‘method’ to distinguish structures, overcoming the lack of commercial markers designed for this taxon.

We identified inflammatory responses characterized by hemorrhagic areas and scar formation, through Coherent anti-Stokes Raman scattering (CARS) and Two-Photon Excited Fluorescence (TPEF). Hemocytes were recognized as major contributor of this response, invading all altered tissues and actively proliferating. Nerve fibers swell and fragment, giving intense endogenous fluorescence signals (TPEF), thus allowing easy recognition of degenerating fibers. Intense regeneration of fibers is observed starting in the central stump and later visible on the opposite side (CARS). The two stumps regenerate toward each other, driven by the connective tissue, as recognized using Second Harmonic Generation signal (SHG).

Our results show that in octopus: (1) Wallerian degeneration occurs in the pallial nerve after lesion, similar to what observed in mammals after axotomy; (2) connective tissue guides axons to re-innervate tissue targets and re-establish the lost function.

## O3 Characterization of *smn*-*1* genetic interactors in a *C.elegans* SMA model

### Pamela Santonicola, Ivan Gallotta, Giuseppina Zampi, Elia Di Schiavi

#### Institute of Bioscience and BioResources, IBBR, CNR, Napoli, Italy

##### **Correspondence:** Elia Di Schiavi (elia.dischiavi@ibbr.cnr.it)

*BMC Neuroscience* 2018, ** 19(suppl 3)**:O3

Spinal muscular atrophy (SMA) is a genetic neuromuscular disorder caused by mutations in the evolutionary conserved Survival of Motor Neurons gene (*SMN1*). The loss of *SMN1* induces a selective degeneration of lower motor neurons (MNs), leading to progressive muscle atrophy and death, but the pathogenesis of the disease and the function played by SMN1 in MNs are still elusive. We used *C.elegans* as an animal model for its powerful genetics and ease of nervous system analysis to study SMN1 in vivo function in MNs survival. We demonstrated that *SMN* orthologue, *smn*-*1,* is functionally conserved and its depletion in 19 ventral MNs causes an alteration in movement, an age-dependent degeneration and neuronal death [1]. We then identified several genes that genetically interact with *smn*-*1* for neuron survival. Interestingly some of these genes are able to prevent the apoptotic death but not the onset of degeneration, while others fully protect neuronal function, integrity and survival. By identifying new modifiers of SMN function we are elucidating the molecular mechanisms that underlie neuron degeneration. Moreover we are using a medium-throughput drug screening to identify small synthetic and natural compounds that revert the neurodegeneration. Since the only treatment recently launched on the market has a limited uptake in many tissues, crucial targets in SMA, and presents obvious clinical challenges, the identification of new molecular targets and new small molecules will be crucial for ameliorating the symptoms of the disease.

**Reference**

1. Gallotta I, Mazzarella N, Donato A, et al. Neuron-specific knock-down of SMN1 causes neuron degeneration and death through an apoptotic mechanism. Hum Mol Genet. 2016; 25: 2564–2577.

## O4 Study of neural stem cell biology in a mouse model of Rett syndrome

### Tiziana Squillaro^1^, Nicola Alessio^2^, Stefania Capasso^2^, Stefania Del Gaudio^2^, Giovanni Di Bernardo^2^, Marilena Cipollaro^2^, Marina AB Melone^1^, Gianfranco Peluso^3^, Umberto Galderisi^2,4^

#### ^1^Department of Medical, Surgical, Neurological, Metabolic Sciences, and Aging; Division of Neurology and InterUniversity Center for Research in Neurosciences, University of Campania “Luigi Vanvitelli”, Napoli, Italy; ^2^Department of Experimental Medicine, University of Campania “Luigi Vanvitelli”, Naples, Italy; ^3^Institute of Agro-Environmental Biology and Forestry (IBAF), CNR, Napoli, Italy; ^4^Sbarro Institute for Cancer Research and Molecular Medicine, Center for Biotechnology, Temple University, Philadelphia, PA, USA

##### **Correspondence:** Tiziana Squillaro (tiziana.squillaro@unicampania.it)

*BMC Neuroscience* 2018, ** 19(suppl 3)**: O4

This abstract is not included here as it has already been published [1].

**Reference**

1. Alessio N, Riccitiello F, Squillaro T, Capasso S, Del Gaudio S, Di Bernardo G, Cipollaro M, Melone MAB, Peluso G, Galderisi U. Neural stem cells from a mouse model of Rett syndrome are prone to senescence, show reduced capacity to cope with genotoxic stress, and are impaired in the differentiation process. Exp Mol Med. 2018; 50(3):1. 10.1038/s12276-017-0005-x.

## O5 Monoacylglycerol lipase inhibition by CPD-4645 reduces diazepam-refractory status epilepticus in mice and its effects are improved by ketogenic diet

### Gaetano Terrone^1,2^, Alberto Pauletti^2^, Alessia Salamone^2^, Massimo Rizzi ^2^, Bianca R Villa^2^, Luca Porcu^3^, Mark J Sheehan^4^, Edward Guilmette^4^, Christopher R Butler^5^, Justin R Piro^4^, Tarek A Samad^4^, Ennio Del Giudice^1^, Annamaria Vezzani^2^

#### ^1^Department of Translational Medicine, Federico II University, Napoli, Italy; ^2^Departments of Neuroscience, IRCCS-Istituto di Ricerche Farmacologiche Mario Negri, Milano, Italy; ^3^ Department of Oncology, IRCCS-Istituto di Ricerche Farmacologiche Mario Negri, Milano, Italy; ^4^Internal Medicine Research Unit, Pfizer Worldwide Research and Development, Cambridge, MA, USA; ^5^Medicinal Chemistry, Pfizer Worldwide Research & Development, Cambridge, MA, USA

##### **Correspondence:** Gaetano Terrone (gaetanoterrone@virgilio.it)

*BMC Neuroscience* 2018, ** 19(suppl 3)**: O5

This abstract is not included here as it has already been published [1].

**Reference**

1. Terrone G, Pauletti A, Salamone A, Rizzi M, Villa BR, Porcu L, Sheehan MJ, Guilmette E, Butler CR, Piro JR, Samad TA, Vezzani A. Inhibition of monoacylglycerol lipase terminates diazepam-resistant status epilepticus in mice and its effects are potentiated by a ketogenic diet. Epilepsia. 2018; 59(1):79-91.

## O6 Orexin and endocannabinoid morphological interactions in the brain of adult zebrafish

### Roberta Imperatore^1,2^, Livia D’Angelo^3^, Giovanni Annona^1^, Nicola Forte^2^, Lea Tunisi^2,3^, Ettore Varricchio^1^, Paolo De Girolamo^3^, Vincenzo Di Marzo^2^, Luigia Cristino^2^, Marina Paolucci^1^

#### ^1^Department of Science and Technology, University of Sannio, Benevento, Italy; ^2^Endocannabinoid Research Group, Institute of Biomolecular Chemistry, CNR, Pozzuoli, Italy; ^3^Department of Veterinary Medicine and Animal Productions, University of Naples Federico II, Napoli, Italy

##### **Correspondence:** Roberta Imperatore (roberta.imperatore@icb.cnr.it)

*BMC Neuroscience* 2018, ** 19(suppl 3)**: O6

This abstract is not included here as it has already been published [1].

**Reference**

1. Imperatore R, D’Angelo L, Safari O, Motlagh HA, Piscitelli F, de Girolamo P, Cristino L, Varricchio E, di Marzo V, Paolucci M. Overlapping Distribution of Orexin and Endocannabinoid Receptors and Their Functional Interaction in the Brain of Adult Zebrafish. Front Neuroanat. 2018; 12:62. 10.3389/fnana.2018.00062.

## O7 Shutting down translation of amyloid precursor protein in early symptomatic hAPP mutant mice rescues the Alzheimer disease phenotype

### Antonella Borreca^1^, Francesco Valeri^2^, Mariassunta A De Luca^2^, Arianna Russo^3^, Stefano Biffo^3^, Alberto Cordella^4,5^, Nicola B Mercuri^5,6^, Martine Ammassari-Teule^1,5^

#### ^1^CNR-IBCN, Roma, Italy; ^2^University of Rome La Sapienza, Roma, Italy; ^3^INGM National Institute of Molecular Genetic, Milano, Italy; ^4^Campus Biomedico-Rome, Roma, Italy; ^5^IRCCS Fondazione Santa Lucia, Roma, Italy; ^6^University of Rome Tor Vergata, Roma, Italy

##### **Correspondence:** Antonella Borreca (antonella.borreca@gmail.com)

*BMC Neuroscience* 2018, ** 19(suppl 3)**: O7

Overexpression of full-length Amyloid Precursor Protein (APP) associates with alterations in APP mRNA translation in Alzheimer Disease (AD) mouse models and sporadic AD patients. Whether these alterations depend on abnormalities in protein synthesis machinery is not well-understood. By performing a polysome gradient analysis in hippocampal samples from hAPPswe mutant mice, we detected a shift of APP mRNA on the polysomal fraction which indicates that the messenger are more prone to be translated before (1 month) or shortly after (3 months) mice start exhibiting AD symptoms. We observed that this shift was associated with decreased phosphorylation of the initial translation factor eIF2α We then found that pharmacological inhibition of eIF2α dephosphorylation in 3-month-old hAPP mice was able to normalize APP levels and to rescue AD hallmarks including Aβ levels, dendritic spine defects, episodic memory deficits, and lack of memory-induced hippocampal *c*-*fos* activation. By identifying an early time-window in which eIF2α phosphorylation pathway is paradoxically downregulated in hAPP mice, our findings reveal that treatments increasing eIF2α phosphorylation which shutdown APP translation within this time-window globally rescues the AD phenotype.

## O8 Protocadherins and their role in brain complexity—a tale from *Octopus*

### Ruth Styfhals^1,2^, Giovanna Ponte^1^, Eve Seuntjens^2^, Graziano Fiorito^1^

#### ^1^Department of Biology and Evolution of Marine Organisms, Stazione Zoologica Anton Dohrn, Napoli, Italy; ^2^Department of Biology - Research group of Developmental Neurobiology, KU Leuven, Leuven, Belgium

##### **Correspondence:** Ruth Styfhals (ruth.styfhals@gmail.com)

*BMC Neuroscience* 2018, ** 19(suppl 3)**: O8

Protocadherins (PCDHs) are transmembrane adhesion molecules that have been implicated in neural wiring, synaptogenesis and neuronal diversity. The expansion of this gene family is seen as a vertebrate innovation and appears essential for creating complex brains. *Drosophila melanogaster* and other invertebrates have a very limited set of PCDHs. In the fruit fly the neuronal diversity is achieved through Dscam proteins (i.e. Down syndrome cell adhesion molecules).

Over 168 protocadherins were found within the genome of *Octopus bimaculoides*, a mollusc, suggesting that it uses a vertebrate-like system to create a complex brain.

We carried out an in silico analysis of the *O. vulgaris* transcriptome and identified putative Ov-Dscam proteins and Ov-PCDHs. By combining a Tblastn analysis with a search for conserved domains using the Pfam database, we improved the currently available annotation. In addition, construction of a phylogenetic tree allowed us to differentiate various isoforms. Subsequently, we analysed RNA-seq expression data of the identified proteins.

We identified a significant number of protocadherins (> 50) in *O. vulgaris* and were able to design specific mRNA probes for in situ hybridizations. We will present our preliminary results of the distribution of protocadherin expression within the *O. vulgaris* brain and will overlay it with recent available maps of the dopaminergic system in the different brain areas. Finally, we will show that only one Ov-Dscam gene is present in the *O. vulgaris* transcriptome, which supports the hypothesis that *O. vulgaris*, a species closely related to *O. bimaculoides*, developed the same system as vertebrates.

## O9 Evidence of increased oxidative stress in Pompe disease. A new therapeutic target?

### Antonietta Tarallo^1,2^, Carla Damiano^1^, Nadia Minopoli^1^, Marcella Coletta^1^, Caterina Porto^1^, Barbara Rossi^2^, Daria M Monti^3^, Giancarlo Parenti^1,2^

#### ^1^Department of Translational Medical Sciences, Federico II University, Napoli, Italy; ^2^Telethon Institute of Genetics and Medicine, Pozzuoli, Italy; ^3^Department of Chemical Sciences, Federico II University, Napoli, Italy

##### **Correspondence:** Antonietta Tarallo (tarallo@tigem.it)

*BMC Neuroscience* 2018, ** 19(suppl 3)**: O9

Pompe disease (PD), or Glycogenosis II, is a hereditary metabolic myopathy caused by the deficiency of acid alpha-glucosidase (GAA). Typical PD histologic abnormalities are the intralysosomal storage of glycogen and the accumulation of autophagic material. It is known that autophagy removes oxygen reactive species (ROS), mitochondria and damaged proteins. It might be speculated that the impairment of the autophagic pathway observed in the PD results in cellular and oxidative stress that may be deleterious for muscle and heart.

To date Enzyme Replacement Therapy (ERT) with human recombinant GAA (rhGAA) is the only pharmacological approach for PD.

We evaluated the level of oxidative stress and its effect on the uptake of rhGAA in fibroblasts of PD patients and in tissues of PD mouse model.

Biochemical tests showed high levels of ROS and lipid peroxidation in PD fibroblasts and in disease-relevant organs form the PD mouse model (heart, quadriceps, gastrocnemius and diaphragm) with respect to the controls. In the same samples we also found reduced glutathione levels and disregulation of the stress (p-ERK, pP38MAPK, Hsp27) and autophagy (LC3, Beclin1) pathways.

By further increasing stress in cells (by starvation or sodium arsenite treatment) we found a reduced efficacy of ERT compared to untreated cells. By using drugs that modulate autophagy or antioxidant we obtained an attenuation of altered markers and improved internalization of rhGAA.

It is reasonable to assume that these secondary abnormalities contribute to the clinical manifestations of PD and affect the efficacy of ERT; therefore, they represent potential therapeutic targets.

## O10 Regulation of trafficking and folding of cellular prion protein PrPC and its shadow Shadoo

### Anna Pepe^1^, Rosario Avolio^1^, Danilo S Matassa^1^, Franca Esposito^1^, Lucio Nitsch^1^, Chiara Zurzolo^2^, Ada Pesapane^3^, Nunzia Montuori^3^, Antonio Lavecchia^4^, Simona Paladino^1,5^, Daniela Sarnataro^1,5^

#### ^1^Department of Molecular Medicine and Medical Biotechnology, University of Naples “Federico II”, 80131, Napoli, Italy; ^2^Institut Pasteur, Unité de Trafic Membranaire et Pathogénèse, 75724 Paris CEDEX 15, France; ^3^Department of Translational Medical Sciences, University of Naples “Federico II”, 80131, Napoli, Italy; ^4^Department of Pharmacy, “Drug Discovery” Laboratory, University of Naples “Federico II”, 80131, Napoli, Italy; ^5^Ceinge-Biotecnologie Avanzate scarl, 80145, Napoli, Italy

##### **Correspondence:** Daniela Sarnataro (sarnatar@unina.it)

*BMC Neuroscience* 2018, ** 19(suppl 3)**: O10

Cellular prion protein (PrPC) is a glycosyl-phosphatidyl-inositol (GPI) anchored glycoprotein whose precise function in the brain remains elusive but may depend upon its cellular localization. PrPC is able to misfold to a pathogenic isoform PrPSc, the causative agent of neurodegenerative prion diseases. The PrPSc formation and cellular propagation has been related to the presence of the PrP receptor 37/67 kDa LR (Laminin Receptor), as well as to a member of the prion protein family called Shadoo. The misfolded PrPSc is amyloidogenic and strictly related to expression, intracellular localization and association of PrPC itself to cholesterol enriched membrane microdomains (lipid rafts).

In this report we show our recent findings related to:

1. The biological effects deriving from the treatment with specific 37/67 kDa LR inhibitor, NSC47924, on the trafficking and interaction between PrPC and its receptor
2. The natural tendency of Shadoo to acquire in neuronal cells “prion-like” characteristics, such as misfolding and aggregation state, and the existence of its non-translocated ER form, whose presence into mitochondria is controlled by the chaperone TRAP1.

Altogether our findings contribute i) to reveal NSC47924 inhibitor as a useful tool to regulate PrPC and 37/67 kDa LR trafficking and degradation, representing a novel small molecule to be tested against prion diseases and ii) to understand the role of molecular chaperones and of PrP-related folding intermediates in “prion-like” conversion.

## O11 Orexin-A enhances dopaminergic signaling in the brain of obese mice

### Lea Tunisi^1,2^, Nicola Forte^1^, Roberta Imperatore^1,3^, Alba C Fernandez-Rilo^1,4^, Isabella Mavaro^1,2^, Livia D’Angelo^2^, Paolo De Girolamo^2^, Letizia Palomba^1,5^, Vincenzo Di Marzo^1,6^, Luigia Cristino^1^

#### ^1^Endocannabinoid Research Group, Institute of Biomolecular Chemistry, CNR, Pozzuoli 80078 Italy; ^2^Department of Veterinary Medicine and Animal Productions, University of Naples Federico II, Napoli 80137 Italy; ^3^Department of Science and Technology, University of Sannio, Benevento 82100 Italy; ^4^University of Campania “Vanvitelli”, Caserta 81100 Italy; ^5^Department of Biomolecular Sciences, University of Urbino, Urbino 61029 Italy; ^6^Canada Excellence Research Chair, Quebec Heart and Lung Institute Research Centre and Institute of Nutrition and Functional Foods, Université Laval, Quebec City, Quebec G1 V 0A6, Canada

##### **Correspondence:** Luigia Cristino (luigia.cristino@icb.cnr.it)

*BMC Neuroscience* 2018, ** 19(suppl 3)**: O11

An endocannabinoid-mediated dishinibition of orexin-A expressing neurons has been described in the hypothalamus of leptin signaling-defective obese mice, such as *ob/ob* or mice fed with high fat diet, (HFD), concurrently with elevation of orexin-A trafficking and release to many target areas. In addition to its role in arousal, the orexin system also has been implicated in reward behaviors since OX-A injection in the ventrotegmental area (VTA) increases dopamine (DA) release in the prefrontal cortex and nucleus accumbens (NAcc) triggering reward-associated behaviors. Here we sought to investigate if aberrant OX-A signaling occurs at VTA DA neurons of obese mice and if it could enhance DA synthesis and release to NAcc and promote reward-associated food seeking behaviors and hyperphagia. With this purpose, by exploiting morphological, biochemical, pharmacological and behavioral approaches we found a significant increase of OX-A release to the VTA of obese mice concomitantly with elevation of DA synthesis and release in the VTA and NAcc. The pharmacological treatment with the OX-1R antagonist SB334867 was able to lower DA levels to control values in the VTA and NAcc of both *ob/ob* and HFD mice. These data suggest that DA synthesis in the VTA and its release to NAcc are modulated by OX-A signaling and are associated with hyperphagia and body weight gain since SB334867 treatment of obese mice reduced body weight. These results are of special relevance since aberrant OX-A signaling during obesity could trigger the vicious circle underlying food seeking reward-associated behaviors, thus contributing to hyperphagia.

## O12 *Onecut* gene function in the CNS of chordates: development and evolution

### Quirino A Vassalli^1^, Chiara Colantuono^2^, Valeria Nittoli^1^, Anna Ferraioli^1^, Giulia Fasano^1^, Maria L Chiusano^2^, Robert Kelsh^3^, Paolo Sordino^1^, Annamaria Locascio^1^

#### ^1^Department of Biology and Evolution of Marine Organisms, Stazione Zoologica Anton Dohrn, Villa Comunale, Napoli, Italy; ^2^Department of Soil, Plant, Environmental and Animal Production Sciences, University of Naples Federico II, Via Università 100, Portici, Italy; ^3^Centre for Regenerative Medicine and Department of Biology and Biochemistry, University of Bath, London, UK

##### **Correspondence:** Annamaria Locascio (annamaria.locascio@szn.it)

*BMC Neuroscience* 2018, ** 19(suppl 3)**: O12

*Onecut* genes (*OC*) have been identified in all major groups of metazoans and are expressed in the nervous system and in some endodermal derived tissues. Their function in liver and pancreas differentiation in mammals has been quite well studied, while almost nothing is known about their function in neurogenesis and eye formation in chordates.

By using the tunicate *Ciona intestinalis* and vertebrate zebrafish *Danio rerio* as model systems, we studied the role and the genetic cascade of *OC* genes in photoreceptor cells and in eye formation during chordate evolution. The *Ciona* genome contains a single *OC* gene, while the analysis of the zebrafish genome revealed the presence of five *OC* orthologues *OC1*, *OC2, OClike* and two gene copies of *OC3*, named *OC3a* and *OC3b*. To acquire novel insights into the degree of *OC* gene functional conservation across chordates, we performed *OC* targeted perturbation by transgenic approach in *Ciona* and *OC* morpholino-mediated knockdown in zebrafish. By differential transcriptomic analyses on *Ciona OC* transgenic embryos we set the ground for the identification of *OC* target genes. The analysis of *OC* morphant phenotypes in zebrafish revealed an *OC* conserved role in the formation of specific eye neural structures. These data insert a new piece in the genetic cascade controlling the specification of the ocellus and eye and highlight a conserved and important role played by *OC* genes in the control of synaptic transmission in various regions of the central nervous system.

## O13 Targeting neuronal proteostasis to treat the CNS in lysosomal storage diseases

### Antonio Monaco, Yulia Ezhova, Teresa Giuliano, Nicolina C Sorrentino, Alessandro Fraldi

#### Telethon Institute of Genetics and Medicine (TIGEM), Napoli, Italy

##### **Correspondence:** Alessandro Fraldi (fraldi@tigem.it)

*BMC Neuroscience* 2018, ** 19(suppl 3)**: O13

Lysosomal storage disorders (LSDs) are severe childhood conditions (incidence: 1/5,000) caused by inherited defects of lysosomal function and often characterized by a neurodegenerative course. There is no cure for the central nervous system (CNS) pathology in these diseases. Understanding the cascade of events consequent to lysosomal dysfunction makes it possible to develop new therapies for LSDs. Data in our laboratory revealed a disease-relevant link between lysosomal dysfunction and defective neuronal proteostasis. Alpha-synuclein and CSP-alpha are two presynaptic chaperones involved in maintaining normal proteostasis at nerve terminals during synaptic activity. By studying mouse models of LSDs we demonstrated that lysosomal dysfunction causes accumulation of alphasynuclein in amyloid aggregates and increased proteasome degradation of CSP-alpha. These events lead to a concurrent loss of these two chaperones at nerve terminals that disrupts presynaptic proteostasis and function, thus initiating neurodegeneration. Building upon these findings we are exploring the possibility to slow down neurodegenerative processes in LSDs reestablishing the physiological levels of CSP-alpha and alpha-synuclein at nerve terminals. Furthermore, characterization of amyloid aggregates showed that these deposits are localized in the perinuclear regions of neurons and in addition to alpha-synuclein contain several other aggregate-prone proteins. We are also evaluating the overall impact of amyloid aggregation on neuropathogenic cascades in LSDs in order to find new therapeutic targets to treat the CNS in these devastating disorders.

## O14 Myoclonic Epilepsy of Unverricht and Lundborg (EPM1): understanding the role of Cystatin b in human cerebral organoids

### Fabrizia Pipicelli^1,2^, Silvia Cappello^2^, Rossella Di Giaimo^1,2^

#### ^1^Department of Biology, University of Napoli, Complesso Universitario Monte S.Angelo, via Cinthia, 80126, Napoli, Italy; ^2^Max Planck Institute of Psychiatry, Kraepelinstraße 2-10, 80804 München, Germany

##### **Correspondence:** Rossella Di Giaimo (digiaimo@unina.it)

*BMC Neuroscience* 2018, ** 19(suppl 3)**: O14

EPM1 is the most common type of progressive myoclonus epilepsy due to different mutations of the Cystatin B (CSTB) gene, such as the null D68 mutation that produces a truncated protein.

CSTB is a widely distributed protein which inhibits proteases of the cysteine family, most commonly Cathepsin B (CTSB).

Preliminary in vivo studies show a high expression of CSTB in radial glial cells during embryonic cortex development and its importance for the maintenance of an appropriate cellular environment, suggesting a non-cell autonomous effect in regulating cell proliferation.

In this study we analyze the role of CSTB in an in vitro model system of human brain development, using human cerebral organoids from induced pluripotent stem cells (IPSC).

The aim is to assess if the overexpression, obtained by electroporation, of CSTB-WT and D68 is able to alter the proliferation of neighboring cells.

Proliferating cells were quantified by immunohistochemistry analysis showing that the overexpression of either CSTB and D68-CSTB causes an alteration of proliferation. Specifically, we detected a significant increase of proliferating cells in CSTB-WT electroporated organoids and a significant reduction of proliferation in CSTB-D68 electroporated organoids.

These results indicate that CSTB has an important function in the niche to maintain an appropriate cellular environment regulating the proliferation of neighboring cells in human cerebral organoids, as in mouse developing cortex.

## O15 A rat model of perinatal stress: impact on gene expression of glutamatergic postsynaptic density genes

### Elisabetta F Buonaguro^1^, Sara Morley-Fletcher^2,3^, Camilla Avagliano^1^, Licia Vellucci^1^, Stefania Maccari^2,4,3^, Andrea de Bartolomeis^1^

#### ^1^University of Naples ‘Federico II’, Laboratory of Molecular and Translational Psychiatry- Department of Neuroscience- Reproductive Sciences and Odontostomatology, Napoli, Italy; ^2^University Lille, Unité de Glycobiologie Structurale et Fonctionnelle, Lille, France; ^3^University Lille, International Associated Laboratory LIA ‘Prenatal Stress and Neurodegenerative Diseases’, Lille, France; ^4^Sapienza University of Rome, IRCCS Neuromed, Roma, Italy

##### **Correspondence:** Camilla Avagliano (camilla.avagliano@gmail.com)

*BMC Neuroscience* 2018, ** 19(suppl 3)**: O15

**Background**

The glutamatergic neurotransmission, particularly the scaffolding family of Homer 1 interacting with mGluR5, is associated with stress psychopathology. It has been observed that perinatal stress may increase the risk for major psychiatric diseases and that both antidepressants and antipsychotics modulate the immediate early gene Homer1a in medial prefrontal cortex.

**Materials and methods**

In perinatally restraint stressed (PRS) rats, i.e., the offspring of dams exposed to repeated episodes of restraint stress during pregnancy, we investigated: (1) light–dark boxes test (LDB) measuring the time and the latency spent in the light compartment; and (2) modifications of expression of genes linked to glutamatergic signaling in different brain regions related to stress response in radioactive in situ hybridization histochemistry.

**Results**

PRS rats showed significant reduction of time spent in the light compartment compared to the control group (*p* < .01), expressing therefore reduced exploratory behavior. PRS rats showed reduced cortical and hippocampal levels of Homer1a and mGluR5. The mRNA levels of the constitutive isoform Homer1b were also tested, with the result of a significant gene expression reduction in the amygdala.

**Conclusions**

Perinatal stress in rats triggers alterations that make adult offspring less resilient to stress, thereby increasing vulnerability to stress-related disorders. The present results highlight an involvement of Homer1a in a perinatal epigenetic animal model of stress and supports the hypothesis of the mGluR5-Homer1 complex as a possible target for new therapeutic approaches.

## O16 Modulation of aging pathways as a therapeutic strategy for Parkinson’s disease

### Federica Esposito^1^, Yolanda Colino-Sanguino^2^, Ivan Gallotta^1,3^, Sean Coakley^4^, Mathilde Faideau^1^, Sandro Montefusco^1^, Elena Polishchuck^1^, Annamaria Carissimo^1^, Anders Bjorklund^3^, Andrea Ballabio^1^, Massimo A Hilliard^4^, Diego Medina^1^, Elia Di Schiavi^2^, Mickael Decressac^1^

#### ^1^Telethon Institute of Genetics and Medicine (TIGEM), Pozzuoli, Italy; ^2^Wallenberg Neuroscience Center, Lund University, Lund, Sweden; ^3^Institute of Biosciences and Bioresources (IBBR), National Research Council, Napoli, Italy; ^4^CJCADR, Queensland Brain Institute, The University of Queensland, Brisbane, Australia

##### **Correspondence:** Mickael Decressac (m.decressac@tigem.it)

*BMC Neuroscience* 2018, ** 19(suppl 3)**: O16

**Background**

Aging is the primary risk factors of the most common neurodegenerative diseases including Parkinson’s disease. In this line, Parkinson’s disease can be seen as a stochastic acceleration or dysfunction of cellular pathways governing cellular senescence. Consequently, a better understanding of the molecular pathways underpinning the aging process can lead to the development of novel therapeutic strategies.

**Material, methods and results**

Using high-content screening, we have identified an evolutionary-conserved signaling pathway that regulates aging. We demonstrate that genetic or pharmacological modulation of this pathway provides neuroprotection and regeneration in in vitro and in vivo models of Parkinson’s disease.

**Conclusions**

Aging manipulation is foremost attractive as a therapeutic approach since it simultaneously targets multiple defensive cellular mechanisms rather than on component of the proteostasis network at a time.

## O17 Alteration of endosomal trafficking is associated with neurodegenerative diseases

### Dominga Fasano^1^, Lucrezia Zerillo^1^, Giovanna M Pierantoni^1^, Anna De Rosa^2^, Marina Picillo^3^, Giuseppina Amodio^4^, Maria T Pellecchia^3^, Paolo Barone^3^, Giuseppe De Michele^2^, Daniela Saranataro^1,5^, Lucio Nitsch^1^, Paolo Remondelli^4^, Chiara Criscuolo^2^, Simona Paladino^1,5^

#### ^1^Dept Molecular Medicine and Medical Biotechnology, University of Naples Federico II, Napoli, Italy; ^2^Dept of Neuroscience, Reproductive, and Odontostomatological Sciences University of Naples Federico II, Napoli, Italy; ^3^Center For Neurodegenerative Diseases (CEMAND), University of Salerno, Salerno, Italy; ^4^Dept of Medicine, Surgery and Dentistry “Scuola Medica Salernitana”, University of Salerno, Salerno, Italy; ^5^CEINGE Biotecnologie Avanzate scarl, Napoli, Italy

##### **Correspondence:** Simona Paladino (spaladin@unina.it)

*BMC Neuroscience* 2018, ** 19(suppl 3)**: O17

Homeostasis of eukaryotic cells is largely dependent on dynamic compartmentalization of the endo-membrane system. The membrane trafficking linking different organelles is essential to maintain a proper composition of various compartments as well as to transport various molecules to appropriate compartments. Respect to other cell types nervous system is more sensitive to alterations of the membrane trafficking. In the last years, we studied the role of endosomal trafficking in neurodegeneration focusing on two nervous system disorders, Charcot-Marie Tooth disease 4 J (CMT4 J) and a new form of autosomal recessive early-onset parkinsonism (PARK20), both caused by mutations of an inositol phosphatase (Fig 4 and Synj1, respectively). Our recent investigations show that:

1. Synj1 plays a crucial role in regulating the homeostasis and functions of early endosomal compartments in different cell types and in fibroblasts of PARK20 patients;
2. the loss of Fig 4 drastically alters the whole endo-lysosome axis (lysosomes, but also late and early endosomes result enlarged and more numerous) implying its essential role for the homeostasis and function of these compartments.

All together, our data provide evidence for the implication of endosomal pathway in neurodegeneration, emphasising the link between endosomal trafficking and neurodegenerative diseases.

## O18 Effects of Methylcyclopentadienyl Manganese Trycarbonil on dopaminergic neurons in zebrafish

### Giulia Fasano^1,2^, Rafael S Godoy^3^, Elisa Angiulli^4^, Ada Consalvo^5,6^, Cristina Franco^1^, Enrico Alleva^7^, Domenico Ciavardelli^5,8^, Mattia Toni^4^, Elio Biffali^2^, Marc Ekker^3^, Paolo Sordino^2^, Lorella MT Canzoniero^1^

#### ^1^Università del Sannio, Benevento, Italy; ^2^Stazione Zoologica Anton Dohrn, Napoli, Italy; ^3^University of Ottawa, Ottawa, Canada; ^4^Sapienza University, Roma, Italy; ^5^CeSI-MeT, Chieti, Italy; ^6^“G. d’Annunzio” University of Chieti-Pescara, Chieti, Italy; ^7^Istituto Superiore di Sanità, Roma, Italy; ^8^“Kore” University of Enna, Enna, Italy

##### **Correspondence:** Paolo Sordino (sordino@szn.it)

*BMC Neuroscience* 2018, ** 19(suppl 3)**: O18

Heavy metals are environmental factors whose role in the pathogenesis of several neurological disorders is taken increasingly into account. Alteration of brain manganese (Mn) homeostasis has been linked to neurodegenerative diseases, such as Alzheimer and Parkinson’s. Strong concern was expressed over Methylcyclopentadienyl Manganese Trycarbonil (MMT), an organic Mn-containing gasoline additive, due to health risk from long-term exposure. Despite evidence of structural and functional damage induced by Mn-based chemicals on dopaminergic (DA) neurons, no in vivo studies on neuronal differentiation have been performed. This study aims to investigate transcriptional, morphological and behavioral alterations caused by sub-lethal (30–100 µM) MMT exposure during neuronal differentiation in zebrafish. Zebrafish is a useful model organism for studying effects of neurotoxicants on the nervous system, by virtue of teleost brain homologies with the mammalian one. Zebrafish embryos exposed to MMT at critical developmental stages of DA differentiation showed transcriptional alteration of genes involved in specification, intermediate differentiation and maturation of DA neurons. MMT treatment was also able to alter morphology, number and size of specific clusters of DA neurons. MMT treatment evoked a hyperactive behavior in embryos. Embryos treated with MMT were grown up to 5 months and submitted to the Y-maze test for evaluating their behavior. The analysis revealed changes in cognitive capabilities affecting exploration, orientation and spatial memory. Collectively, these findings suggest that chronic exposure to sub-lethal MMT during neuronal differentiation can alter the development of DA neurons and the short- and long-term behavioural traits controlled by the DA system.

## O19 The inflammatory response following acute seizures in zebrafish brain and in chordate evolution

### Valeria Nittoli^1^, Alessandra Gentile^2^, Giulia Fasano^1^, Antonio Palladino^1^, Marco Borra^1^, Paolo De Girolamo^3^, Antonietta Spagnuolo^1^, Paolo Sordino^1^

#### ^1^Stazione Zoologica Anton Dohrn, Napoli, Italy; ^2^MPI- HLR, Bad Nauheim, Germany; ^3^Università degli Studi di Napoli Federico II, Napoli, Italy

##### **Correspondence:** Paolo Sordino (sordino@szn.it)

*BMC Neuroscience* 2018, ** 19(suppl 3)**: O19

Inflammation represents the natural body defense to various types of insults and is a part of a complex cascade of events, closely linked to the activation of the immune system. Although inflammation is linked to repair processes, it can be detrimental if dysregulated. Recently, clinical and experimental findings have highlighted the importance of inflammation in epilepsy, supporting an important role for inflammatory mediators in seizure activity, and suggesting the beneficial effects of anti-inflammatory medications for some refractory epilepsy forms. In this context, the discovery of new molecules targeting inflammatory pathways gives the opportunity to enrich the repertoire of drugs counteracting epileptic seizures. To this aim, we adopt the zebrafish model of seizure induced by pro-convulsive agent, the pentylenetetrazole (PTZ), to analyze the expression dynamic of inflammatory molecules and cells, and investigated the functional role of some inflammatory pathways. Moreover, to explore the degree of phylogenetic conservation of the seizure-related inflammatory mechanisms among chordates, we are investigating other emerging model systems such as the tunicate *Ciona robusta*. PTZ-induced seizures in zebrafish are associated with an increase of key inflammatory molecules that accompanies the activation of microglial cells and with a rapid induction of neuroprotective mechanisms. The genetic manipulation and pharmacological treatments strongly support the hypothesis of a direct role of some inflammatory pathways in the seizure activity. Our findings provide further therapeutic targets for the high-throughput usage of zebrafish in the discovery of anti-inflammatory drugs. Furthermore, preliminary data suggest the use of *C. robusta* to explore seizure-induced inflammatory response during evolution.

**POSTERS**

## P1 Serotonin 5-HT7 receptor increases the density of dendritic spines and facilitates synaptogenesis in forebrain neurons

### Luisa Speranza^1^, Floriana Volpicelli^1^, Salvatore Pulcrano^1^, Marianna Crispino^2^, Giancarlo Bellenchi^1^, Umberto di Porzio^1^, Carla Perrone-Capano^1,3^

#### ^1^Institute of Genetics and Biophysics “Adriano Buzzati Traverso”, CNR, Napoli, Italy; ^2^Department of Biology, University of Naples Federico II, Napoli, Italy; ^3^Department of Pharmacy, University of Bari “A. Moro”, Bari, Italy

##### **Correspondence:** Carla Perrone-Capano (perrone@unina.it)

*BMC Neuroscience* 2018, ** 19(suppl 3)**: P1

In normal and pathological brain functions the precise control of dendritic spine density and synapse formation is critical. Therefore, how signaling pathways influence dendrite outgrowth and remodeling is still difficult to define. Here, we report that prolonged activation of the serotonin 5-HT7 receptor (5-HT7R) with a selective agonist promotes formation of dendritic spines and facilitates synaptogenesis in postnatal cortical and striatal neurons. Acute activation of 5-HT7R results in pronounced neurite elongation in postnatal striatal and cortical neurons, thus extending previous data on the morphogenic role of 5-HT7R in embryonic neurons. Critical role of 5-HT7R in neuronal morphogenesis was confirmed by analysis of neurons isolated from 5-HT7R-deficient mice and by pharmacological inactivation of the receptor. In addition, we also observed decreased number of spines when 5-HT7R was blocked pharmacologically, and in 5-HT7R-knock-out neurons, suggesting that constitutive 5-HT7R activity is critically involved in the spinogenesis. Moreover, cyclin-dependent kinase 5 and small GTPase Cdc42 were identified as important downstream effectors mediating morphogenic effects of 5-HT7R in neurons. Altogether, our data suggest that the 5-HT7R-mediated structural reorganization during the postnatal development might have a crucial role for the development and plasticity of forebrain areas such as cortex and striatum, and thereby can be implicated in regulation of the higher cognitive functions. Future analyses will be addressed to identify specific miRNAs involved in remodeling of dendritic spines and synaptogenesis. Understanding the way miRNAs are contributing to synaptic plasticity could provide clues to establish novel therapeutic strategies for several diseases of the Nervous System.

## P2 Modulation of synaptic protein synthesis in animal model of Alzheimer’s disease

### Carmela Barbato^1^, Carolina Cefaliello^1^, Eduardo Penna^1^, Giuseppina Di Ruberto^1^, Carla Perrone-Capano^2^, Maria C Miniaci^2^, Marianna Crispino^1^

#### ^1^Department of Biology, University of Naples Federico II, Napoli, Italy; ^2^Department of Pharmacy, University of Naples Federico II, Napoli, Italy

##### **Correspondence:** Marianna Crispino (crispino@unina.it)

*BMC Neuroscience* 2018, ** 19(suppl 3)**: P2

A local system of protein synthesis, independent and remote from the cell body, is present in the presynaptic domains of axons. The prompt synthesis of protein on site and on demand near the synapses makes key contributions to synaptic plasticity. Nonetheless, not much has been explored whether its deregulation leads to the neurodegenerative diseases. In this study, we examine the involvement of the synaptic system of protein synthesis in the pathogenesis of Alzheimer’s disease (AD), using transgenic mice overexpressing APP as animal model (TG). To this end, we have tested if the local system of protein synthesis is modulated by contextual fear conditioning, and if such modulation is deregulated in the TG animals. We prepared synaptosomes as an in vitro model for synaptic regions. The synaptosomal fractions from cerebral cortex and cerebellum were incubated with a radio-labelled amino acid and the pattern of newly synthetized proteins was analyzed. In the wild type animals, the newly-synthesized proteins were modulated by training. By contrast, this modulation did not occur in the TG animals, suggesting that the synaptic system of protein synthesis is impaired in AD mice. These results indicate that the synaptic protein synthesis may also be involved in the molecular mechanism leading to synaptic degeneration in AD.

## P3 A *C.elegans* model to study LDL-related proteins involvement in Alzheimer’s disease

### Carla Bertapelle^1,2^, Alessandro Medoro^1^, Federica Cocco^1^, Elia Di Schiavi^2^, Claudio Russo^1^

#### ^1^Department of Health Sciences, University of Molise, Campobasso, Italy; ^2^Institute of Bioscience and BioResources, CNR, Napoli, Italy

##### **Correspondence:** Elia Di Schiavi (elia.dischiavi@ibbr.cnr.it)

*BMC Neuroscience* 2018, ** 19(suppl 3)**: P3

The Alzheimer’s disease (AD) is the most common neurodegenerative disorder and its neuropathologic hallmarks are the extracellular deposits of β-amyloid (Aβ) plaques and intraneuronal tangles, resulting from Tau hyperphosphorylation. These aggregates both contribute to the loss of synapse functions and neuronal death. The major genetic risk factor in sporadic AD is represented by the E4 isoform of ApolipoproteinE (ApoE), ligand of the Low Density Lipoprotein-Related Proteins Receptor 8 (LRP8). LRPs processing is regulated by γ-secretase cleavage in a similar way to the Amyloid Precursor Protein (*APP*), which after cleavage by γ-secretase produces the Aβ peptide. Considering the lack of informations about LRPs involvement in AD, we propose a *C.elegans* model to study in vivo the correlation of human LRP8 to APP and Tau.

*C.elegans* presents orthologs of APP, γ-secretase and Tau (*apl*-*1*, *sel*-*12* and *ptl*-*1*), and it has been extensively used to study AD. We generated multiple transgenic lines overexpressing in all neurons the human LRP8 protein. By Western blot we verified the expression of LRP8 protein as full-length and cleaved forms, resembling the peptides observed in cell cultures and AD patients. The transgenic lines present defects in locomotion, development and lifespan in a similar way to other *C.elegans* AD models. In order to verify if LRP8 is cleaved by γ-secretase ortholog, we are inhibiting the protease activity using both a pharmacological and a genetic approach. We set up a new model, which may help clarify the role of LRPs in AD, and its involvement in regulating APP and Tau.

## P4 A functional study of the endocannabinoid system in zebrafish neurodevelopment: implications in vision and locomotion

### Rosa M Sepe^1^, Raffaella De Paolo^1^, Jean-Michel Cioni^2^, Jingjing Zang^3^, Stephan CF Neuhauss^3^, William A Di Marzo^5^, Paolo Sordino^1^

#### ^1^Biology and Evolution of Marine Organisms (BEOM), Stazione Zoologica Anton Dohrn, Napoli, Italy; ^2^Department of Physiology, Development and Neuroscience, University of Cambridge, Cambridge CB2 3DY, UK; ^3^Institute of Molecular Life Sciences, University of Zurich, Zurich, Switzerland; ^4^Dipartimento di Medicina veterinaria e Produzioni animali, University of Naples “Federico II”, Napoli, Italy; ^5^Endocannabinoid Research Group, Institute of Biomolecular Chemistry, Consiglio Nazionale delle Ricerche, Pozzuoli, Italy

##### **Correspondence:** Paolo Sordino (sordino@szn.it)

*BMC Neuroscience* 2018, ** 19(suppl 3)**: P4

The endocannabinoid system (ECS) comprises neuromodulatory lipids and their receptors, capable of regulating neuronal excitability, and recently, it has been suggested that the ECS may play an important role in early neuronal development. The arachidonoylglycerol (2-AG) is synthesized at high levels in the CNS by diacylglycerol lipase α (*Daglα*). Our aim was to investigate the role of 2-AG and its receptors (CB1 and CB2) in the development and differentiation of neurons, and in the formation of neuronal circuits that control spontaneous locomotion and visual system, using zebrafish as model organism. Through the use of morpholino-induced transient knockdown of the zebrafish *daglα* and its pharmacological rescue, we suggest that synthesis of 2-AG is implicated in the control of axon formation in defined areas of the developing brain, and animals lacking Daglα display defective axonal growth and fasciculation, and abnormal physiological behavior in tests measuring stereotyped eye movement and motion perception. Furthermore, pharmacological treatments using antagonists for CB1 and CB2 highlight their role in the correct differentiation and lamination of zebrafish developing retina. Animals treated with these antagonists display also defective swimming behavior, suggesting the implication of CB1 and CB2 receptors also in the correct formation of neuronal circuits that control spontaneous locomotion. In conclusion, our results suggest an important role of endocannabinoids as mediators in axonal outgrowth with implications for the control of vision and movement.

## P5 The LRRK2-R1441C mutation disrupts long-term potentiation-like plasticity in Parkinson’s disease patients

### Marika Ranieri, Raffaele Dubbioso, Anna De Rosa, Marcello Esposito, Silvio Peluso, Rosa Iodice, Giuseppe De Michele, Lucio Santoro, Fiore Manganelli

#### Department of Neurosciences, Reproductive Sciences and Odontostomatology, Federico II University of Naples, Napoli, Italy

##### **Correspondence:** Raffaele Dubbioso (rafdubbioso@gmail.com)

*BMC Neuroscience* 2018, ** 19(suppl 3)**: P5

This abstract is not included here as it has already been published [1].

**Reference**

1. Dubbioso R, de Rosa A, Esposito M, Peluso S, Iodice R, de Michele G, Santoro L, Manganelli F. Does motor cortex plasticity depend on the type of mutation in the leucine-rich repeat kinase 2 gene? Mov Disord. 2017; 32(6): 947–948.

## P6 Corpus callosum abnormalities: neuroimaging, cytogenetics and clinical characterization of a very large multicenter Italian series

### Giuseppina Vitiello^1^, Romina Romaniello^2^, Alessandra D’Amico^3^, Mariasavina Severino^4^, Filippo Arrigoni^5^, Rita Genesio^6^, Floriana Imperati^1^, Orsetta Zuffardi^7^, The Italian CCA Study Group (Achille Iolascon, Nicola Brunetti-Pierri, Alfonso Romano, Carmela Bravaccio, Marco Carotenuto, Daniela Melis, Gaetano Terrone, Piero Pignataro, Lorenzo Ugga, Arturo Brunetti, Vincenzo Nigro, Angela Francesca Crisanti, Edoardo Errichiello, Giovanni Cioni, Simona Fiori, Paola Brovedani, Daria Riva, Nardo Nardocci, Stefano D’Arrigo, Luisa Chiapparini, Livia Garavelli, Carmine Pascarella, Ivan Ivanovski, Vincenzo Leuzzi, Alberto Spalice, Mario Mastrangelo, Luigi Tarani, Francesco Nicita, Giacomo Garone, Mariachiara Colaiacomo, Claudio Di Biasi, Pasquale Parisi, Alessandro Ferretti, Francesco Brancati, Francesco Garaci, Federica Carla Sangiuolo, Federico Vigevano, Marina Trivisano, Enricosilvio Bertini, Lorena Travaglini, Giangennaro Coppola, Lucia Margari, Francesca Operto, Mattia Gentile, Elisa Franzoni, Duccio Maria Cordelli, Francesco Toni, Veronica Di Pisa, Giuseppe Gobbi, Lucia Marangio, Margherita Santucci, Monica Maffei, Melissa Filippini, Anna Ficcadenti, Gabriele Polonara, Nelia Zamboni, Sabrina Siliquini, Claudia Passamonti, Agata Fiumara, Simone Palmucci, Rita Barone, Elisa Fazzi, Patrizia Accorsi, Paola Martelli, Lucio Giordano, Serena Micheletti, Lorenzo Pinelli, Pasquale Striano, Valeria Capra, Sara Uccella, Margherita Mancardi, Edvige Veneselli, Michela Sole, Francesca Maria Battaglia, Agnese Suppiej, Elisa Cainelli, Giacomo Talenti, Giuseppe Sartori, Irene Toldo, Eleonora Lorenzon, Angelo Selicorni, Silvia Maitz), Lucio Nitsch^5^, Renato Borgatti ^2^, Ennio Del Giudice^1^

#### ^1^Department of Translational Medicine, Section of Pediatrics, Federico II University, Naples, Italy; ^2^Neuropsychiatry and Neurorehabilitation Unit, IRCCS Eugenio Medea, Bosisio Parini, Lecco, Italy; ^3^Department of Advanced Biomedical Sciences, Federico II University, Naples, Italy; ^4^Neuroradiology Unit, Istituto Giannina Gaslini, Genoa, Italy; ^5^Neuroimaging Laboratory, E. Medea Research Institute, Bosisio Parini, Italy; ^6^Department of Molecular Medicine and Medical Biotechnologies, Federico II University, Naples, Italy; ^7^Department of Molecular Medicine, University of Pavia, Pavia, Italy

##### **Correspondence:** Ennio Del Giudice (endelgiu@unina.it)

*BMC Neuroscience* 2018, ** 19(suppl 3)**: P6

Corpus callosum abnormalities (CCA) have an estimated prevalence ranging from 0.3% up to 0.7% in patients undergoing brain imaging. CCA can be identified incidentally, or can be part of a developmental disease. We performed a retrospective study of 551 patients, identified non-syndromic (NS) CCA and syndromic (S) CCA, reviewing clinical features, neuroradiological aspects, genetic etiology, and chromosomal microarray (CMA) results. Syndromic CCA subjects were prevalent (60%) and they showed the most severe clinical features. Cortical malformations and cerebellar anomalies were 23% of cerebral malformation associated to CCA (plus), 23 and 14% respectively in syndromic forms. A clinical and/or genetic diagnosis was obtained in 37% of syndromic CCA including chromosomal rearrangements on high-resolution karyotype (18%), microdeletion/microduplication syndromes (31%) and monogenic diseases (51%). Non-syndromic CCA anomalies had mildest clinical features, although intellectual disability was present in 49% of cases and epilepsy in 13%. CMA diagnostic rate in our cohort of patients ranged from 11 to 23% (NS to S). A high percentage of patients (76% 422/551) remain without a diagnosis. Combined high resolution CMA studies and next-generation sequencing (NGS) strategies will increase the probability to identify new causative genes of CCA and to redefine genotype–phenotype correlation.

## P7 EPM1: understanding the role of Cystatin b in the mouse developing cortex

### Martina Giordano^1^, Christina Kyrousi^1^, Silvia Cappello^1^, Rossella Di Giaimo^1,2^

#### ^1^Max Planck Institute of Psychiatry, Kraepelinstraße 2-10, 80804 München, Germany; ^2^Department of Biology, University of Napoli, Complesso Universitario Monte S.Angelo, via Cinthia, 80126, Napoli, Italy

##### **Correspondence:** Rossella Di Giaimo (digiaimo@unina.it)

*BMC Neuroscience* 2018, ** 19(suppl 3)**: P7

Progressive myoclonus epilepsy of the Unverricht–Lundborg-type (EPM1) is an autosomal recessive neurodegenerative disorder and it is the most common type of progressive myoclonus epilepsy.

The physiological function of CYSTATIN-B (CSTB) in the CNS and the dysfunction caused by the mutants are still unknown but loss of-function mutations in the gene encoding CSTB are the primary genetic cause of EPM1. CSTB-KO mice show neurological disorder in mice similar to EPM1-patients. Mutations which lead to epilepsy often develop during embryogenesis, for this reason we decided to study the role of CSTB by interfering with its expression in the mouse developing cortex.

To this aim, we overexpressed CSTB wild type and 2 human pathological mutants, G4R and D68, by in utero electroporation, in mouse developing cortex at E14 and we analysed the results 2 days later. Our results show different distribution of electroporated cells. Cells overexpressing wtCSTB at 2dpe migrated from VZ to IZ while cells electroporated with the 2 mutants are located only in the VZ, indicating a difference in cell migration and/or differentiation.

In addition, using different markers for cell proliferation, we detected an increase of proliferation in the region surrounding the wtCSTB-electroporated cortical area. On the contrary we detected a decrease in proliferation in the cortex upon overexpression of the 2 EPM1 mutants. Our results clearly indicate a neurogenic role of CSTB during cortical development, which is altered by overexpression of the protein or by the expression of an EPM1-mutant form.

## P8 Four-year electrophysiological and neuropsychological follow-up in the adult form of Niemann Pick disease type C

### Giulia Mele, Stefano Tozza, Raffaele Dubbioso, Rosa Iodice, Antonietta Topa, Marcello Esposito, Lucia Ruggiero, Emanuele Spina, Aniello Iovino, Lucio Santoro, Fiore Manganelli

#### Department of Neurosciences, Reproductive Sciences and Odontostomatology, Federico II University of Naples, Napoli, Italy

##### **Correspondence:** Raffaele Dubbioso (rafdubbioso@gmail.com)

*BMC Neuroscience* 2018, ** 19(suppl 3)**: P8

This abstract is not included here as it has already been published [1].

**Reference**

1. Tozza S, Dubbioso R, Iodice R, Topa A, Esposito M, Ruggiero L, Spina E, De Rosa A, Saccà F, Santoro L, Manganelli F. Long-term therapy with miglustat and cognitive decline in the adult form of Niemann-Pick disease type C: a case report. Neurol Sci. 2018; 39(6):1015–1019

## P9 Beneficial effects of curcumin on peripheral symptoms of Huntington’s disease

### Elena Montano^1^, Salvatore Castaldo^2^, Francesca Elifani^2^, Viola Calabrò^1^, Tiziana Angrisano^1^, Vittorio Maglione^2^, Alba Di Pardo^2^, Alessandra Pollice^1^

#### ^1^Department of Biology, University of Naples Federico II, Napoli, Italy; ^2^IRCCS Neuromed Centre for Neurogenetics and Rare Diseases, Pozzilli, Isernia, Italy

##### **Correspondence:** Alessandra Pollice (apollice@unina.it)

*BMC Neuroscience* 2018, ** 19(suppl 3)**: P9

Huntington’s disease (HD) is a genetic neurodegenerative disorder characterized by a complex pathogenic and clinical profile with neuronal dysfunction and progressive atrophy in the striatum and in the cerebral cortex. Aside defects in the nervous system, the disease is characterized by a variety of peripheral complications among which progressive weight loss that correlates with disease progression and significantly affects the quality of life of HD patients. The mechanism underlying weight loss is unknown but several evidences suggest that it may likely be attributable to structural and functional alteration of the intestinal tract. Although efforts have been made in HD research, no effective treatment to counteract any of the symptoms is currently available. Over the last years utilization of natural products like curcumin, have had large diffusion for the treatments of neurodegenerative diseases, however much remains to be explored yet. The aim of our study it to investigate the molecular mechanism underlying alterations in the intestinal tract potentially linked to the weight loss in HD and to explore the therapeutic potential of curcumin administration in R6/2 mice, which represent the best characterized and most widely used animal model of HD that recapitulate most of the features of the human pathology.

## P10 Characterisation in *C. elegans* of mutations in DAT gene causative of dopamine transporter deficiency syndrome

### Maria Paglione^1^, Ambra Lanzo^1^, Giuseppina Zampi^1^, Manju Kurian^2^, Elia Di Schiavi^1^

#### ^1^Institute of Biosciences and BioResources, IBBR, CNR, Napoli, Italy; ^2^UCL Institute of Child Health, Molecular Neurosciences, Developmental Neurosciences, UCL Great Ormond Street Institute of Child Health, 30 Guilford Street, London WC1 N 1EH, UK

##### **Correspondence:** Elia Di Schiavi (elia.dischiavi@ibbr.cnr.it)

*BMC Neuroscience* 2018, ** 19(suppl 3)**: P10

Dopamine transporter deficiency syndrome (DTDS) is an autosomal recessive disorder caused by mutations in the human gene encoding for the dopamine transporter (*hDAT*), which leads to the partial or total loss of function of the protein. The DTDS phenotypic spectrum is characterized by a typical form, with early onset parkinsonism-like symptomatology, that was distinguished from atypical forms presenting milder conditions and caused by partial loss of protein function. The aim of this work is to investigate in vivo the effects of the new genomic variants found in DAT gene in patients, using *Caenorhabditis elegans*. *hDAT* is *cedat*-*1* in *C.elegans* and its knockout causes a locomotion defect due to a lack of reuptake of dopamine and resistance to 6-hydroxydopamine toxic effects. We established the first animal model for DTDS and demonstrated that *hDAT* is able to rescue the two phenotypes observed when *cedat*-*1* is knocked out. To examine the effects on *DAT* function of two mutations recently found in patients, we expressed the human mutated forms *hDAT*^*G433R*^ or *hDAT*^*G467V*^ only in dopaminergic neurons in *cedat*-*1* KO and studied the possible rescue of the defective phenotypes. Transgenic animals expressing *hDAT*^*G467V*^ showed a partial rescue of only one of the two phenotypes, whereas the *hDAT*^*G433R*^ mutant showed impairment in DAT function in both phenotypic paradigms. Taken together, these observations suggest that *G433R* mutation causes the complete loss of function in vivo, while the *G467V* mutation causes a partial loss of protein function, resembling other mutations causing atypical forms of DTDS.

## P11 Cystatin B secretion in adult mammalian brains

### Eduardo Penna^1^, Fabrizia Pipicelli^1^, Angela Cerciello^1^, Rossella Di Giaimo^1,2^, Marianna Crispino^1^

#### ^1^Department of Biology, University of Naples Federico II, Napoli, Italy; ^2^Max Planck Institute of Psychiatry, Kraepelinstraße 2-10, 80804 München, Germany

##### **Correspondence:** Rossella Di Giaimo, Marianna Crispino (digiaimo@unina.it; marianna.crispino@unina.it)

*BMC Neuroscience* 2018, ** 19(suppl 3)**: P11

Cystatin B (CSTB) is a widely distributed protein expressed in most cell types and tissues where it inhibits proteases of the cysteine family. Mutation of CSTB causes Progressive Myoclonus Epilepsy (EPM1), a disease of the Central Nervous System, suggesting a key role of this protein in the physiology of the Nervous System. Previous studies of our group showed that CSTB is secreted during brain development, as CSTB was found in the cerebrospinal fluid (CSF) from the developing brain of mouse. In addition, the culture medium of primary cortical cells transfected with a plasmid expressing the CSTB-GFP fusion protein contained endogenous CSTB as well as the CSTB-GFP protein. Interestingly, transfection of a natural EPM1 mutant did not result in CSTB secretion suggesting that secretion of the protein is critical for its function.

Here we detected the presence of CSTB in the synaptic regions of adult rodents brain, using synaptosomes isolated from brain cortex as in vitro model of nerve endings. Synaptosomes are an useful tool to study the function of synaptic terminals, such as secretion. After 2 h incubation of synaptosomes CSTB was detected in medium, indicating an active secretion of this protein from the nerve endings of adult rodents brain. In addition, we found CSTB in a crude synaptosomal fraction prepared from human cerebral organoids. Its expression level increased with the maturation of the organoids, suggesting that CSTB is indicative of progressive synaptogenesis in organoids which can be used as an innovative tool to study neuronal plasticity in a human model.

## P12 Disruption of GABA(A)-mediated intracortical inhibition in patients with Chorea-acanthocytosis

### Giuseppina Ciccarelli, Raffaele Dubbioso, Marcello Esposito, Silvio Peluso, Rosa Iodice, Giuseppe De Michele, Lucio Santoro, Fiore Manganelli

#### Department of Neurosciences, Reproductive Sciences and Odontostomatology, Federico II University of Naples, Napoli, Italy

##### **Correspondence:** Raffaele Dubbioso (rafdubbioso@gmail.com)

*BMC Neuroscience* 2018, ** 19(suppl 3)**: P12

This abstract is not included here as it has already been published [1].

**Reference**

1. Dubbioso R, Esposito M, Peluso S, Iodice R, De Michele G, Santoro L, Manganelli F. Disruption of GABA(A)-mediated intracortical inhibition in patients with chorea-acanthocytosis. Neurosci Lett. 2017; 654:107–110.

## P13 Go-opsin expressing cells in sea urchin larvae produce a TRH neuropeptide, which influences arm growth

### Maria Cocurullo^1^, Natalie Wood^2^, Paola Oliveri^2^, Maria I Arnone^1^

#### ^1^Stazione Zoologica Anton Dohrn, Napoli, Italy; ^2^University College London, London, UK

##### **Correspondence:** Maria I Arnone (miarnone@szn.it)

*BMC Neuroscience* 2018, ** 19(suppl 3)**: P13

In mammals, the Thyrotropin-Releasing Hormone (TRH) has a central role on regulation of metabolism and growth by stimulating the secretion of the TSH from the pituitary gland. In non-mammalian vertebrates, e.g. amphibians and fish, TRH also regulate growth by stimulating the release of Growth hormone and prolactin from the pituitary gland, while it has little or no effect on the secretion of TSH. The role of TRH so far remains poorly investigated in invertebrates. Recently, Van Sinay et al. [1] identified a TRH-like peptide in *C. elegans* and investigated its role in this protostome worm. This study shows that TRH originated before the divergence of protostomes and deuterostomes and suggests that the ancestral role of this neuropeptide is on the control of postembryonic growth and reproduction.

Combining in situ hybridisation, whole-mouth immunostaining and knock-down experiments we investigated the role of a sea urchin TRH neuropeptide (QYPGamide) its precursor (SpTRH) and potential receptor (SpTRHR) in *Strongylocentrotus purpuratus* and *Paracentrotus lividus* larvae. In sea urchins, SpTRH is produced by two go-opsin expressing cells bilaterally distributed at each the side of the larval apical organ. Our data shows that TRH protein production is regulated by light/dark cycle and feeding/starving conditions. Furthermore, knock-down experiments of the SpTRH precursor and of the potential receptor SpTRHR inhibit the post embryonic arm growth.

In conclusion, our results describe for the first time the role of TRH in a non-chordate deuterostomes and validate the hypothesis of an ancestral role of TRH on postembryonic growth regulation.

**Reference**

1. Van Sinay E, Mirabeau O, Depuydt G, et al. Evolutionarily conserved TRH neuropeptide pathway regulates growth in Caenorhabditis elegans. *Proc Natl Acad Sci* 2017; 114: E4065–E4074.

## P14 The experience of the “Federico II” unit of the Telethon Undiagnosed Diseases Program

### Michele Pinelli^1,2^, Gerarda Cappuccio^1,2^, Marianna Alagia^1,2^, Annalaura Torella^1,3^, Simona Fecarotta^2^, Giancarlo Parenti^1,2^, Francesco Musacchia^1^, Margherita Mutarelli^1^, Raffaele Castello^1^, Sandro Banfi^1,3^, Giorgio Casari^1,4^, Vincenzo Nigro^1,3^, Nicola Brunetti-Pierri^1,2^ and TUDP

#### ^1^Telethon Institute of Genetics and Medicine (TIGEM), Pozzuoli, Italy; ^2^Department of Translational Medicine, Federico II University, Naples, Italy; ^3^Medical Genetics, Department of Biochemistry, Biophysics and General Pathology, University of Campania ‘Luigi Vanvitelli’, Naples, Italy; ^4^Università San Raffaele, Milano, Italy

##### **Correspondence:** Michele Pinelli (m.pinelli@tigem.it)

*BMC Neuroscience* 2018, ** 19(suppl 3)**: P14

We present the experience of the “Federico II” unit of the Telethon Undiagnosed Diseases Program (TUDP). TUDP is aimed at the diagnosis of children with rare and severe diseases that lack a genetic diagnosis. TUDP is a national program founded by Fondazione Telethon and is open to all patients, and to clinicians following patients, with undiagnosed diseases. The general principle of the study is to offer an accurate clinical re-evaluation of all patients and, if indicated, a Next Generation Sequencing (NGS) test.

Ours is the referring clinical center of TUDP for the southern and central Italy. From March 2016 to September 2017, we have enrolled 51 pediatric patients with severe, multisystem and undiagnosed diseases. Most of those patients were already been visited by multiple centers and subject to invasive and non-invasive procedures. All were already tested with array-CGH.

Conclusive genetic diagnosis was reached for 11 cases, candidate-gene mutations for 7 cases, and no potential explanation for 4 cases. Out the remaining, 13 cases were considered low-priority and, thus, not addressed to NGS and 16 are still in progress.

Among the concluded cases: one had a mutation in a novel disease-causing gene and its pathogenetic role has been confirmed in other cases; two had phenotypes more complex and severe than those described in literature; most of the remaining cases had clinical presentations too aspecific to prompt a specific genetic test and their diagnosis would have unlikely been reached without an NGS approach.

## P15 Molecular and morphological study of adult neurogenesis in the short-lived fish *Nothobranchius furzeri*

### Adele Leggieri^1,2^, Livia D’Angelo^1^, Luciana Castaldo^1^, Carla Lucini^1^, Paolo de Girolamo^1^, Alessandro Cellerino^2,3^, Luigi Avallone^1^

#### ^1^Department of Veterinary Medicine and Animal Productions, University of Naples “Federico II” – Napoli, Italy; ^2^Leibniz Institute on Aging, Fritz Lipmann Institute – Jena, Germany; ^3^Scuola Normale Superiore di Pisa, Pisa, Italy

##### **Correspondence:** Adele Leggieri (adele.leggieri@unina.it)

*BMC Neuroscience* 2018, ** 19(suppl 3)**: P15

Adult neurogenesis is a dynamic and highly regulated process, decreasing exponentially with aging. In mammals it is spatially restricted into two specific regions: the subventricular and the subgranular zone. In contrast to mammals, teleost fish show an extensive adult neurogenesis along the whole rostro-caudal brain axis. Among teleost fish, *Nothobranchius furzeri* is a well consolidated model organism for ageing research, for its short lifespan and rapid growth. Clustering temporal profile of gene expression in *N. furzeri* brain, have shown differentially expressed genes (DEGs), most of which positively selected during aging and evolution. Here, we investigate the neurogenic activity of three positively selected genes: Alpha Chain 1 Collagen type IV (Col4a1) and XXV (Col25a1), and Inhibitor of DNA Binding 3 (Id3). Whole brain quantitative analysis on young (5 weeks post hatching, wph) and old (27 wph) specimens, have shown a slightly upregulation of these genes during aging. Fluorescence in situ hybridization (FISH) on both young and old animals revealed that Col4a1, Col25a1 and Id3’s mRNAs are distributed in neurogenic areas. Furthermore, their distribution pattern changes according to growth. Then, to describe the different developing stages of FISH positive cells, we performed a double labeling of FISH and immunofluorescence (anti- S100, anti-DCX, and anti-HuC/D). Genes and neural markers co-localize in most of adult neurogenic areas.

These studies suggest that Col4a1, Col25a1 and Id3 are involved in neurogenic process during aging, as already shown in many species. Our findings could form the basis for further investigations on their specific roles and pathways.

## P16 Variables affecting pain threshold evaluation in autoalgometry

### Letizia Lorusso, Andrea Viggiano

#### Università degli Studi di Salerno, Dip. Medicina, Chirurgia e Odontoiatria “Scuola Medica Salernitana”, Salerno, Italy

##### **Correspondence:** Andrea Viggiano (aviggiano@unisa.it)

*BMC Neuroscience* 2018, ** 19(suppl 3)**: P16

Pain threshold is the measure of the intensity of a physical stimulus that evokes pain. To avoid the influence of the “tester”, a new method has been recently proposed, named “autoalgometry”, in which the subject being evaluated applies and controls by himself the force against the autoalgometer tip.

The aim of the present work was to evaluate the effects of stimulation rate, gender and site of stimulation on the pain threshold evaluated in healthy subjects. Fifty healthy volunteers (21 males, 29 females, age 18–29 years) were evaluated for the pain threshold using the autoalgometric procedure by applying fast- or slow-increasing stimulation on a computerized autoalgometer tip with their fingers and reaching a minimal or maximal pain intensity.

There was a positive correlation between test speed and pain threshold measures. Male participants reached higher speeds compared to female participants when asked to execute fast and showed higher pain thresholds (both for the minimal and the maximal pain intensity) compared to female participants in the fast tests. When the tests were executed slowly, the minimal pain threshold did not differ between males and females, but the maximal pain threshold was still higher in males compared to females.

These results demonstrate that it is mandatory to record the rate of stimulation in a pressure pain-threshold evaluation and support the use of the autoalgometric procedure for this purpose.

## P17 Inflammasome and autophagy cross-talk in bovine brains: preliminary observations

### Davide De Biase^1^, Claudio Pirozzi^2^, Giuseppe Piegari^1^, Teresa B Pagano^1^, Francesco Prisco^1^, Giuseppina Mattace Raso^1^, Serenella Papparella^1^, Orlando Paciello^1^

#### ^1^Department of Veterinary Medicine and Animal Production, University of Naples Federico II, via Delpino 1, 80137, Napoli, Italy; ^2^Department of Pharmacy, University of Naples Federico II, Via Montesano 49, 80131 Napoli, Italy

##### **Correspondence:** Davide De Biase (davide.debiase@unina.it)

*BMC Neuroscience* 2018, ** 19(suppl 3)**: P17

*Inflammaging* occurs in the aged brain both as microglia senescence and as an increased production of pro-inflammatory cytokines and protein complexes known as *inflammasomes*. Recently, it has been suggested that autophagy acts as a regulator of NLRP3 inflammasome activation. Here, we describe our findings concerning the expression of MHC II as a marker of microglia senescence and NLRP3 inflammasome in bovine brains and the cross-talk between inflammasome, autophagy and ROS production. Samples of hippocampus were collected from 42 Podolica cattle. Animals were divided in three groups: group A (aged 15–24 years), group B (aged 5–14 years) and group C (aged up to 5 years). Double color immunofluorescence and immunohistochemistry were performed to evaluate 1) the relationship between NLRP3, SOD1 and autophagy marker Beclin 1 and 2) the expression of MHC II and NLRP3. Western blot was performed to determine the expression levels of NLRP3. Double color immunofluorescence showed a positive relationship between inflammasome, Beclin 1 and SOD1. Moreover, immunoistochemistry revealed a higher expression of MHC II and NLRP3 in older animals. Western blot confirmed the higher expression of NLRP3 in aged bovine compared to younger. These data demonstrate that MHC II and NLRP3 are up-regulated in the brain of aged cattle. We suggest that the age-related overexpression of SOD1 indicates an excessive production of ROS resulting in deleterious peroxidative reactions and ultimately in neuroinflammation. Moreover, we propose that autophagy may protect the cell by removing inflammasome components from the cell, thus maintaining cellular homeostasis.

